# Patients with Allan‐Herndon‐Dudley Syndrome (MCT8 Deficiency) Display Symptoms of Parkinsonism in Childhood and Respond to Levodopa/Carbidopa Treatment

**DOI:** 10.1002/mds.30152

**Published:** 2025-03-15

**Authors:** Nina‐Maria Wilpert, Angela L. Hewitt, Roser Pons, Marie‐Thérèse Henke, Andrea Dell'Orco, Martin Bauer, Christiane Grolik, Stephan Menz, Monika Wahle, Annika Zink, Alessandro Prigione, Christina Reinauer, Catharina Lange, Christian Furth, Knut Brockmann, Sabine Jung‐Klawitter, Stine Christ, Angela M. Kaindl, Anna Tietze, Heiko Krude, Thomas Opladen, Markus Schuelke

**Affiliations:** ^1^ Department of Neuropediatrics Charité‐Universitätsmedizin Berlin, Corporate member of Freie Universität Berlin, Humboldt‐Universität zu Berlin, and Berlin Institute of Health (BIH) Berlin Germany; ^2^ Center for Chronically Sick Children Charité‐Universitätsmedizin Berlin, Corporate member of Freie Universität Berlin, Humboldt‐Universität zu Berlin, and Berlin Institute of Health (BIH) Berlin Germany; ^3^ NeuroCure Cluster of Excellence Charité‐Universitätsmedizin Berlin, Corporate member of Freie Universität Berlin, Humboldt‐Universität zu Berlin, and Berlin Institute of Health (BIH) Berlin Germany; ^4^ BIH Charité Junior Clinician Scientist Program Berlin Institute of Health at Charité‐Universitätsmedizin Berlin, BIH Biomedical Innovation Academy Berlin Germany; ^5^ Division of Movement Disorders, Department of Neurology University of Rochester Medical Center, Motor Physiology and Neuromodulation Program Rochester New York USA; ^6^ Division of Child Neurology, Department of Neurology University of Rochester Medical Center Rochester New York USA; ^7^ First Department of Pediatrics National and Kapodistrian University of Athens, Hospital Agia Sofía, Pediatric Neurology Unit Athens Greece; ^8^ Institute of Neuroradiology Charité‐Universitätsmedizin Berlin, Corporate member of Freie Universität Berlin, Humboldt‐Universität zu Berlin, and Berlin Institute of Health (BIH) Berlin Germany; ^9^ Physikalisch‐Technische Bundesanstalt (PTB) Braunschweig Germany; ^10^ Department of Neurology Charité‐Universitätsmedizin Berlin, Corporate member of Freie Universität Berlin, Humboldt‐Universität zu Berlin, and Berlin Institute of Health (BIH) Berlin Germany; ^11^ Institute of Medical Psychology Charité‐Universitätsmedizin Berlin, Corporate member of Freie Universität Berlin, Humboldt‐Universität zu Berlin, and Berlin Institute of Health (BIH) Berlin Germany; ^12^ Department of Pediatrics, Pediatric Neurology Kinderkrankenhaus Amsterdamer Strasse, Kliniken der Stadt Köln Cologne Germany; ^13^ Department of General Pediatrics, Neonatology and Pediatric Cardiology Medical Faculty and University Hospital Düsseldorf, Heinrich Heine University Düsseldorf Germany; ^14^ Department of Nuclear Medicine Charité‐Universitätsmedizin Berlin, Corporate member of Freie Universität Berlin, Humboldt‐Universität zu Berlin, and Berlin Institute of Health (BIH) Berlin Germany; ^15^ Department of Pediatrics and Adolescent Medicine University Medical Center Göttingen Germany; ^16^ Department I, Division of Pediatric Neurology and Metabolic Medicine Heidelberg University, Medical Faculty Heidelberg, Center for Pediatric and Adolescent Medicine Heidelberg Germany; ^17^ Institute for Cell and Neurobiology Charité‐Universitätsmedizin Berlin, Corporate member of Freie Universität Berlin, Humboldt‐Universität zu Berlin, and Berlin Institute of Health (BIH) Berlin Germany; ^18^ partner site Berlin German Center for Child and Adolescent Health (DZKJ), Section CNS Development and Neurological Disease Berlin Germany; ^19^ Institute of Experimental Pediatric Endocrinology Charité‐Universitätsmedizin Berlin, Corporate member of Freie Universität Berlin, Humboldt‐Universität zu Berlin, and Berlin Institute of Health (BIH) Berlin Germany

**Keywords:** MCT8, *SLC16A2*, movement disorder, parkinsonism, dopamine, neurodevelopment

## Abstract

**Background:**

Patients with mutations in the monocarboxylate transporter 8 (MCT8, *SLC16A2*) suffer from X‐linked recessive Allan‐Herndon‐Dudley syndrome (AHDS), which is characterized by developmental delay and a severe movement disorder. Current trials using thyroid hormone derivatives to overcome the transporter defect have failed to achieve patient‐oriented therapeutic goals.

**Objectives:**

Our aim was to define the type of movement disorder in AHDS in an observational cohort study and to investigate the causative role of the dopaminergic system.

**Methods:**

We present longitudinal clinical data from the DEEPTYPE registry of 11 patients with video documentation, standardized phenotyping, cerebrospinal fluid (CSF) analysis, neuroimaging data, and the treatment response to levodopa/carbidopa supplementation.

**Results:**

Children presented with signs of childhood parkinsonism, including hypokinesia, hypomimia, inability to sit or stand, rigidity, dystonia, and autonomic dysfunction. CSF homovanillic acid concentrations were decreased (n = 12), suggesting an isolated dopamine pathway impairment. Seven out of 8 patients responded favorably to l‐dopa/carbidopa supplementation and we did not observe any adverse drug reactions.

**Conclusions:**

AHDS is associated with childhood parkinsonism, which is linked with biochemical abnormalities of dopamine metabolism. It can be treated with l‐dopa/carbidopa supplementation. However, further research is needed to elucidate the exact effect of MCT8 deficiency on dopamine metabolism. © 2025 The Author(s). *Movement Disorders* published by Wiley Periodicals LLC on behalf of International Parkinson and Movement Disorder Society.

Allan‐Herndon‐Dudley syndrome (AHDS) is an X‐linked recessively inherited disability syndrome, first described in 1944.^1^ Additional symptoms such as “dysarthria”, “athetosis”, “extrapyramidal symptoms”, “muscular hypotonia”, and “severe motor developmental delay” were described as clinical features of the disease. In 2004, researchers discovered mutations in the *SLC16A2* gene on the X chromosome as the cause of AHDS. This gene encodes the thyroid hormone transporter monocarboxylate transporter 8 (MCT8).^2^, ^3^ As MCT8 deficiency does not affect thyroid hormone synthesis, patients have normal to elevated triiodothyronine (T3), and thyroid‐stimulating hormone (TSH), as well as normal to decreased thyroxine (T4) serum concentrations (Fig. 1).^4^ Due to the transporter defect, thyroid hormone is unable to reach its intracellular targets in the brain^5^ leading to a state of local (central) hypothyroidism.^6^

**FIG. 1:**
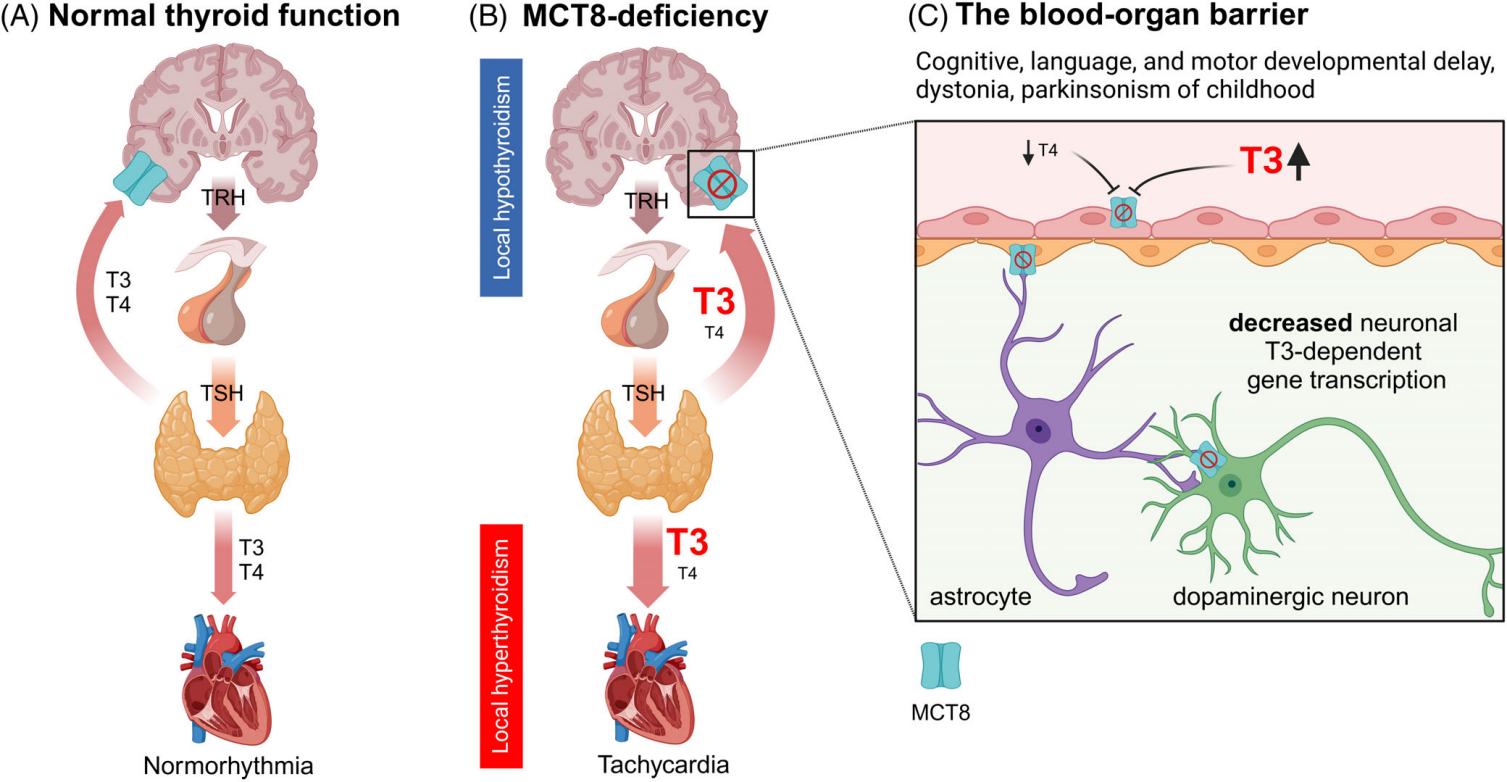
Schematic and phenotypic representation of MCT8 deficiency. (**A, B**) In MCT8 deficiency, thyroid hormones are released from the thyroid gland into the bloodstream with increased triiodothyronine (T3) and normal to decreased thyroxine (T4) concentrations. However, due to inactivating mutations of the *SLC16A2* gene, which encodes the thyroid hormone transporter MCT8 (depicted in blue), T3 and T4 cannot be transported across blood‐organ barriers that depend on MCT8, such as the (**C**) neurovascular unit (endothelial cells, red; pericytes, orange; astrocytes, purple; neurons, green). The brain is subsequently hypothyroid in the prenatal and in the postnatal phases. In other organs that express alternative thyroid hormone transporters, increased T3 concentrations can reach their intracellular thyroid hormone receptors and lead to hyperthyroid states, such as tachycardia. TRH, thyrotropin releasing hormone; TSH, thyroid stimulating hormone. The figure was created with BioRender.com. [Color figure can be viewed at wileyonlinelibrary.com]

The symptom severity of AHDS patients varies from severe phenotypes functionally similar to children with dyskinetic cerebral palsy (with Gross Motor Function Classification System Grade V, GMFCS V) to milder phenotypes with varying developmental delays. Patients can be diagnosed by routine diagnostic exome sequencing and analysis of the fT3/fT4 ratio (http://www.thyroid-hormone-ratio.org).^4^

Only recently, the neurological symptoms, especially the associated movement disorder, have received more attention,^7^, ^8^, ^9^ which constitutes the main disease burden for patients and their caregivers.^10^ Therapeutic approaches comprise the supplementation of patients with thyroid hormone analogues such as tiratricol (Triac) and diiodothyropropionic acid (DITPA), which can bypass the transport by MCT8 *in vitro*,^11^, ^12^ but did not substantially improve clinical outcomes in patients. While reducing elevated peripheral T3 concentrations, the study drugs failed to achieve patient‐oriented therapeutic goals such as improvement in gross, fine motor, and verbal skills, and a reduction in “muscle cramps/stiffness.”^10^

As most of the neurological symptoms relate to the movement disorder, therapies such as l‐dopa/carbidopa supplementation could be an alternative strategy, until suitable gene therapy becomes available.^13^, ^14^ Here we present a retrospective longitudinal study of a larger group of patients with this ultra‐rare disease, documented by videos and motor symptom scoring. In addition, we measured cerebrospinal fluid (CSF) concentrations of neurotransmitters in n = 12 patients and followed n = 6 patients with regular clinical scoring before and after treatment with l‐dopa/carbidopa for up to 12 months. We demonstrate that parkinsonism as part of AHDS is treatable.

## Patients and Methods

### Patient Enrollment and Follow‐Up

Patients and their families were recruited into the DEEPTYPE registry study (https://clinicaltrials.gov/study/NCT06566066) for patients with thyroid hormone resistance between July 2021 and July 2024 from different centers in Germany. All patients carried a hemizygous mutation in *SLC16A2*. Patients' caregivers provided written informed consent according to the Declaration of Helsinki for participation in the study and for publication of results and patient images. The deep phenotyping study was observational only and did not influence treatment decisions. Data were collected in a REDCap registry. Ethical approval for the study was granted by the Institutional Review Board of Charité (EA2/026/20).

During regular visits of our patients, we recorded videos according to the protocol of the Dyskinesia Impairment Scale^15^ and phenotyped the patients at regular intervals applying the following scales: Burke‐Fahn‐Marsden Dystonia Rating Scale (BFMDRS),^16^ Infantile Parkinsonism‐Dystonia Rating Scale (IPDRS),^17^ (Data S1), Bayley Scales of Infant and Toddler Development III (BSID‐III),^18^ Cerebral Palsy Child Health Index of Life with Disabilities,^19^ which are relevant for the evaluation of disease progression and treatment response. Patients were evaluated by a multidisciplinary team of pediatric neurologists, psychologists, physical, occupational, and speech therapists. The BFMDRS was modified by excluding items assessing swallowing function because of its inability to differentiate dysphagia of different etiologies, as described elsewhere.^7^ The presence of dystonia and parkinsonism required a lumbar puncture to test for dopamine deficiency. CSF was flash frozen in liquid nitrogen and analyzed for biogenic amine, 5‐methyltetrahydrofolate (5‐MTHF), and pterin concentrations with minor modifications as described.^20^ Six patients received l‐dopa/carbidopa 4:1 suspension orally or *via* gastric tube. Medication was started at 1.00/0.25 mg/kg/day in four doses per day under inpatient supervision for at least 4 days. Subsequently, the dose was increased by 1.00/0.25 mg/kg/day per week depending on the clinical response up to a maximum dose of 10.0/2.5 mg/kg/day in all children.

### Neuroimaging

Neuroimaging data were also collected retrospectively, the details of which are listed in Data S2. Briefly, for volumetric neuroimaging, MRI images were segmented according to the Desikan‐Killiany Atlas^21^ using Freesurfer version 6.0.^22^ 18F‐DOPA positron emission tomography (PET) data were recorded from patients 8 and 10 and compared to normal values.^23^

### Statistical Analysis

Statistical differences and significance levels between groups were determined using GraphPad Prism 10 (GraphPad Software) with various statistical tests indicated in the respective figure legends. A *P*‐value of <0.05 was considered statistically significant. Results are presented as scatter plots, with bars representing the mean and standard deviation (SD). For natural history data, we attempted to contact the corresponding authors of each study or digitized data from published figures using ImageJ.

## Results

### Patients Present with Signs of Parkinsonism in Childhood

We collected retrospective data from n = 11 male patients with AHDS (age range: 1.0–25.5 years, mean: 7.3 years) (Table 1). All had fT3/fT4 ratios above the 97th percentile^4^ and carried pathogenic *SLC16A2* mutations, inherited from an unaffected mother in 63% (5 of 8) or arising *de novo* in 37% (3 of 8) of the patients.

**TABLE 1:** Clinical description of the cohort

Patient no.	Age [y]	*SLC16A2* mutation [NM_006517.5]	MCT8	ClinVar Accession	ACMG variant classification (pathogenicity)	Inheritance	AHDS pheno‐type	Infan‐tile onset	Hypo kinesia	Rigidity	Dystonia	Axial hypo‐tonia	Brady kinesia	Failure to develop a postural reaction	Glabellar reflex	Low threshold startle response	Response to levodopa/ carbidopa
1	1.0	c.220_222 delinsTT	p.(Val74Phefs*10)	Not reported before	PVSs (very strong)	X‐linked	Severe	+	+	−	+	+	+	+	+	+	Not treated
2	2.0	c.590G>A	p.(Arg197His)	SCV000203572, SCV001572983, SCV004033317, SCV004300116	PS1, PS3 (strong)	X‐linked	Severe	+	+	(+)	+	+	+	+	+	+	+
3	2.1	c.1399G>A	p.(Gly467Ser)	Not reported before	PS2, PM5 (strong)	*de novo*	Moderate	+	+	(+)	+	+	−	+	+	−	+
4	3.7	c.1171‐1G>T	not investigated	Not reported before	Possible PVS1 (very strong)	X‐linked	Severe	+	+	+	+	+	+	+	+	+	+
5	4.9	c.852_862dup GCCCAGCTCCC	p.(Gln288Argfs*59)	SCV002032376	PVS1 (very strong)	X‐linked	Severe	+	+	+	+	+	+	+	+	+	+
6	5.5	c.1439 T>C	p.(Phe480Ser)	not reported before	PM5 (moderate)	Not investigated	Severe	+	+	+	+	+	+	+	na	+	Not treated
7	6.1	pathogenic mutation in *SLC16A2* (§)	na	na	na	Not investigated	Severe	+	+	−	+	+	+	+	+	+	+
8	6.9	c.1235dupG	p.(Leu413Profs*25)	not reported before	PVS1 (very strong)	X‐linked	Severe	+	+	+	+	+	+	+	+	+	+
9	7.4	c.1378A>T	p.(Ile460Phe)	not reported before	PS2 (strong)	*de novo*	Mild	+	−	−	+	−	−	−	−	−	Mildly affected, therefore not treated
10	15.4	c.979G>A	p.(Gly327Arg)	SCV000194929, SCV000492794, SCV001446448, SCV001582829	PS2 (strong)	*de novo*	Severe	+	+	+	+	+	+	+	+	+	+
11	25.5	c.437_440dup AGCG	p.(Val148Alafs*47)	not reported before	PVS1 (very strong)	Not investigated	Severe	+	na	−	+	+	+	+	na	na	Treated for only one week without response

The table depicts the patients' geno‐ and phenotypes. If already reported before, we provide the ClinVar accession numbers for the respective mutations. The variant classification was done according to the guidelines of the American College of Medical Genetics (ACMG).^46^ Age, current age; +, positive; (+), slightly positive; −, negative; na, not assessed; §, the molecular diagnosis had been established some time ago by a commercial laboratory (Ambry Genetics, Aliso Viejo, CA, USA), which only reported the presence of a pathogenic variant in *SLC16A2*, but not its identity.

Abbreviations: AHDS, Allan‐Herndon‐Dudley syndrome; ACMG, The American College of Medical Genetics and Genomics.

Consistent with published phenotypic data,^7^, ^24^ the AHDS phenotype varied in severity, with most patients (82%, 9 of 11) presenting with severe global developmental delay and movement disorder, functionally similar to dyskinetic cerebral palsy with GMFCS V. According to the classification of Leuzzi and colleagues,^25^ these severely affected patients showed signs and symptoms of parkinsonism in childhood with infantile onset. In all cases, head control did not develop during the first months of life. Further symptoms comprised hypokinesia (hypomimia, reduced spontaneous and voluntary movements), bradykinesia, rigidity (independent of a task or posture), dystonia, severe axial hypotonia (head lag and trunk hypotonia), autonomic dysfunction (excessive diaphoresis, obstipation, audible obstructive breathing, sleep disturbance), and motor developmental delay (Video 1; for a detailed list see Table 1). Whereas the hypokinesia, hypotonia, autonomic dysfunction, and developmental delay were present just after birth, we observed the rigidity and dystonia to be increasing with age. Parents did not observe diurnal fluctuations. On glabellar reflex testing, the patients were unable to resist blinking (positive Myerson's sign), and their startle response was strikingly pronounced. Oculogyric crises were neither described by the parents nor observed during clinical examination.

**Video 1:** Patients with Allan‐Herndon‐Dudley syndrome present symptoms of parkinsonism in childhood.

A 2.1‐year‐old patient (patient 3) was classified as moderately affected with global developmental delay (head control at 10 months, free sitting at 12 months, pulling to a standing position at 18 months, and walking along objects at 24 months), suspected intellectual disability and functionally disabling dystonia and hypokinesia (GMFCS II). Another 7.4‐year‐old patient (patient 9) was classified as mildly affected with mild intellectual disability and infrequent action‐specific dystonia of the limbs (GMFCS I) (videos of the last two cases are available at reference [4]).

### Altered Biogenic Amine Concentrations Indicate an Isolated Dopamine Pathway Impairment

**FIG. 2:**
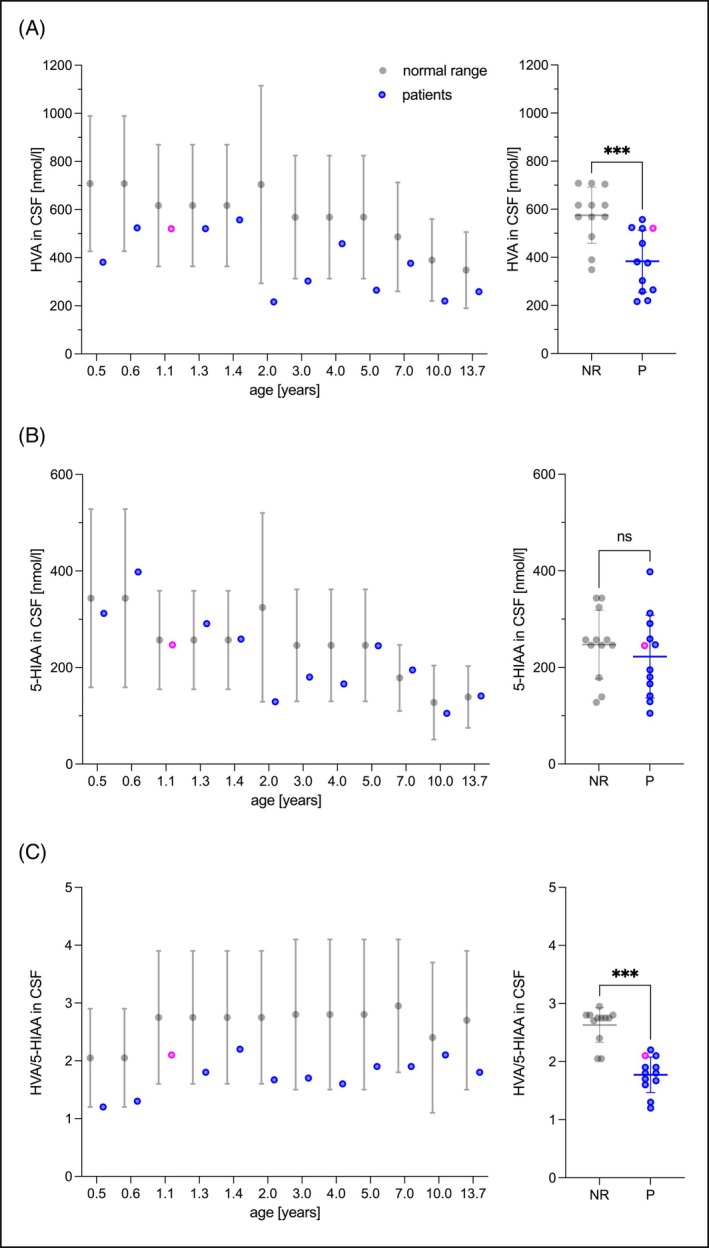
Biogenic amines in the cerebrospinal fluid (CSF). (**A**) The homovanillic acid (HVA) concentrations in the CSF were below the reference ranges (depicted in gray) in 42% (5 of 12) of patients (depicted in blue). In the remaining 58% (7 of 12) of cases, HVA concentrations were observed to fall within the two lower quartiles of the normal range. In general, the HVA concentrations of patients were markedly lower than the mean of the reference data. Patient 1 (depicted in pink) had undergone treatment with Triac (97 μg/kg/day) for 6 months prior to the lumbar puncture. However, the biogenic amine concentrations in the CSF of this patient were comparable to those of untreated children. (**B**) The 5‐hydroxyindoleacetic acid (5‐HIAA) concentrations were within the reference range, and (**C**) the HVA/5‐HIAA ratios were significantly reduced in patients. This constellation of biogenic amine alterations indicates an isolated defect of dopamine deficiency, as observed in tyrosine hydroxylase deficiency, albeit with less pronounced HVA decreases. (**A, B**) The normality of the distribution was tested with the Shapiro–Wilk test (*α* = 0.05). The statistical significance of the differences between the groups was analyzed by applying a paired *t* test. (B) If the data were not normally distributed, the statistical significance of the differences between the groups was tested by applying the Wilcoxon test. ns, not significant; ***P ≤ 0.001; NR, normal range; P, patients. [Color figure can be viewed at wileyonlinelibrary.com]

Biogenic amines, 5‐MTHF, and pterins were measured in n = 12 CSF samples from n = 11 patients (Fig. 2). Patient 10 underwent lumbar puncture at age 3 during the initial clinical evaluation and later at age 13.7 years because of suspected parkinsonism in childhood. Although 5‐MTHF and pterin levels were normal, biogenic amine analyses revealed significantly lower CSF homovanillic acid (HVA) concentrations in AHDS patients with a mean difference of −191.8 nmol/L compared to normal age‐matched controls (*P* < 0.001) (Fig. 2A). However, CSF HVA concentrations were reduced to an average of 67% of normal HVA concentrations, not as markedly reduced as would be expected for a primary dopamine synthesis deficiency as seen in tyrosine hydroxylase deficiency.^26^ The 5‐hydroxyindoleacetic acid (5‐HIAA) concentrations were normal (Fig. 2B), and consequently the HVA/5‐HIAA ratio was significantly reduced in the CSF of AHDS patients with a median difference of −0.9 (*P* < 0.001) (Fig. 2C). We did not find any changes in 3‐*O*‐methyldopa, l‐dopa, or 5‐hydroxytryptophan concentrations. In conclusion, the constellation of biogenic amine alterations in the CSF of AHDS patients strongly suggests an isolated impairment of the dopamine pathway, consistent with the symptoms of parkinsonism in childhood.^25^

### Patients Persistently Respond to l‐Dopa/Carbidopa Treatment

We initiated l‐dopa/carbidopa treatment in n = 6 patients at Charité‐Universitätsmedizin Berlin because of suspected parkinsonism in childhood. Only 2 patients were not treated, 1 (patient 9) with a rare and functionally irrelevant dystonia and 1 patient (patient 6) who was lost to follow‐up. The l‐dopa/carbidopa dosage was slowly increased to 10.0/2.5 mg/kg/day over 10 weeks in all patients. All parents reported improvement in patient‐oriented outcomes.^10^ About 100% (all of 6) had improvement in expressive language (syllables), 83% (5 of 6) had improvement in gross motor skills (head control, first steps) and improved targeted movements (reaching for objects, selective opening and closing of hands), 67% (4 of 6) had less muscle “cramps/stiffness,” and 50% (3 of 6) had improved dysphagia (less hypersalivation and choking on food intake) (data not shown, Video 2).

**Video 2:** Clinical improvement under levodopa/carbidopa therapy.

**FIG. 3:**
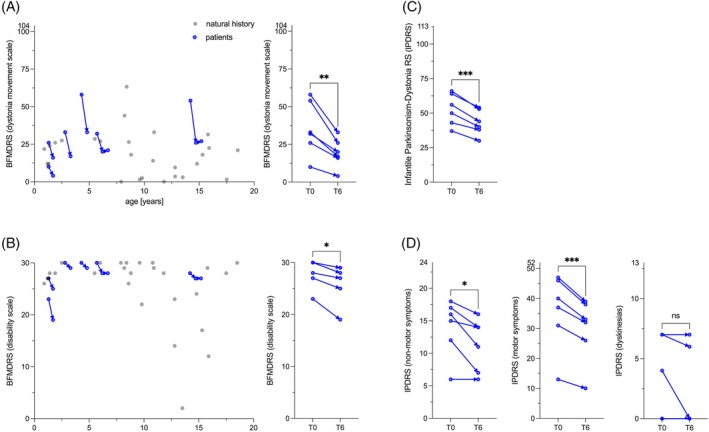
Patients' responses to levodopa/carbidopa therapy. (**A**) Patients (depicted in blue) exhibited a notable improvement in dystonic movement and (**B**) overall disability, as evaluated by the Burke‐Fahn‐Marsden Dystonia Rating Scale (BFMDRS). The natural history data (depicted in gray) were taken from previously published work, and the BFMDRS was modified by excluding items pertaining to swallowing abilities in accordance with the requisite modifications.^7^ (**C**) Additionally, the patients exhibited significantly lower ratings on the Infantile Parkinsonism‐Dystonia Rating Scale (IPDRS) following 6 months of l‐dopa/carbidopa treatment. This was particularly evident in the reduction of non‐motor symptoms, including autonomic dysfunction and mood dysregulation, as well as motor signs of parkinsonism and dystonia, such as hypokinesia, rigidity, dystonia, axial hypotonia, and motor developmental delay. (**A–D**) The normality of the distributions was assessed using the Shapiro–Wilk test (*α* = 0.05). The statistical significance of the differences between the groups was evaluated through the application of a paired *t* test. The following symbols were used to indicate the level of significance: ns, not significant; *P ≤ 0.05; **P ≤ 0.01; ***P ≤ 0.001. T0 represents the baseline measurement, whereas T6 denotes the assessment conducted after 6 months of l‐dopa/carbidopa therapy. [Color figure can be viewed at wileyonlinelibrary.com]

Parental impressions were validated using the BFMDRS, the IPDRS, and the BSID‐III. The dystonia movement subscale of the BFMDRS improved significantly in all patients with a mean difference of −16.2 points (*P* < 0.01) at 6 months and in 2 patients with sustained improvement after 12 months of treatment (Fig. 3A). The disability subscale of the BFMDRS improved less, but also significantly, with a mean difference of −1.8 points (*P* < 0.05) (Fig. 3B). In addition, the total score of the IPDRS improved significantly by −9.5 points (*P* < 0.001) (Fig. 3C). Within the subcategories, patients showed significant improvement in parent‐reported non‐motor signs (autonomic, mood dysfunction) (*P* < 0.05), and investigator‐rated motor signs of parkinsonism and dystonia (hypokinesia, rigidity, dystonia, axial hypotonia, motor developmental delay) (*P* < 0.001) (Fig. 3D). No oculogyric crisis was ever observed. Myoclonus was present in 50% (3 of 6) of the patients but did not improve with therapy.

Consistent with the parents' perception of improvement in goal‐directed movements, patients had significantly higher age equivalents by 1.2 months on the BSID‐III fine motor subscale (*P* < 0.05) (Fig. S1B). In our small cohort, we did not observe any significant improvement in gross motor skills, expressive/receptive language, or cognition (Fig. S2A,C–E). However, none of the patients showed developmental regression, but rather a trend toward improvement. The CPCHILD did not improve. One family (patient 4) even reported a drastic decrease in quality of life due to multiple surgeries on their child (placement of a gastric tube and orthopedic hip surgery) (Fig. S1F). Under l‐dopa/carbidopa therapy, the patients did not experience any adverse drug reactions or laboratory abnormalities (Fig. S2B–F). The z score of the body weight (compared to healthy children) decreased significantly by 0.2 points over the 6 months (*P* < 0.05), which was, however, within the body weight trajectory of the entire cohort of MCT8‐deficient patients (Fig. S2A). The z score of the body mass index, however, did not change significantly in the l‐dopa/carbidopa treated patients. Patient 7 received l‐dopa/carbidopa at the University of Rochester Medical Center up to 3.00/0.75 mg/kg/day for 3 years. The mother reported “improved facial expression, axial tone, less fisting of the hands, better reaching out to objects and longer head control” after having started l‐dopa/carbidopa therapy. Patient 11 received levodopa/carbidopa at the Georg‐August‐University Göttingen up to 9.5/2.4 mg/kg/day for only 1 week and showed no signs of improvement.

### Patients with MCT8 Deficiency Have a Reduced Subcortical Gray Matter Volume

**FIG. 4:**
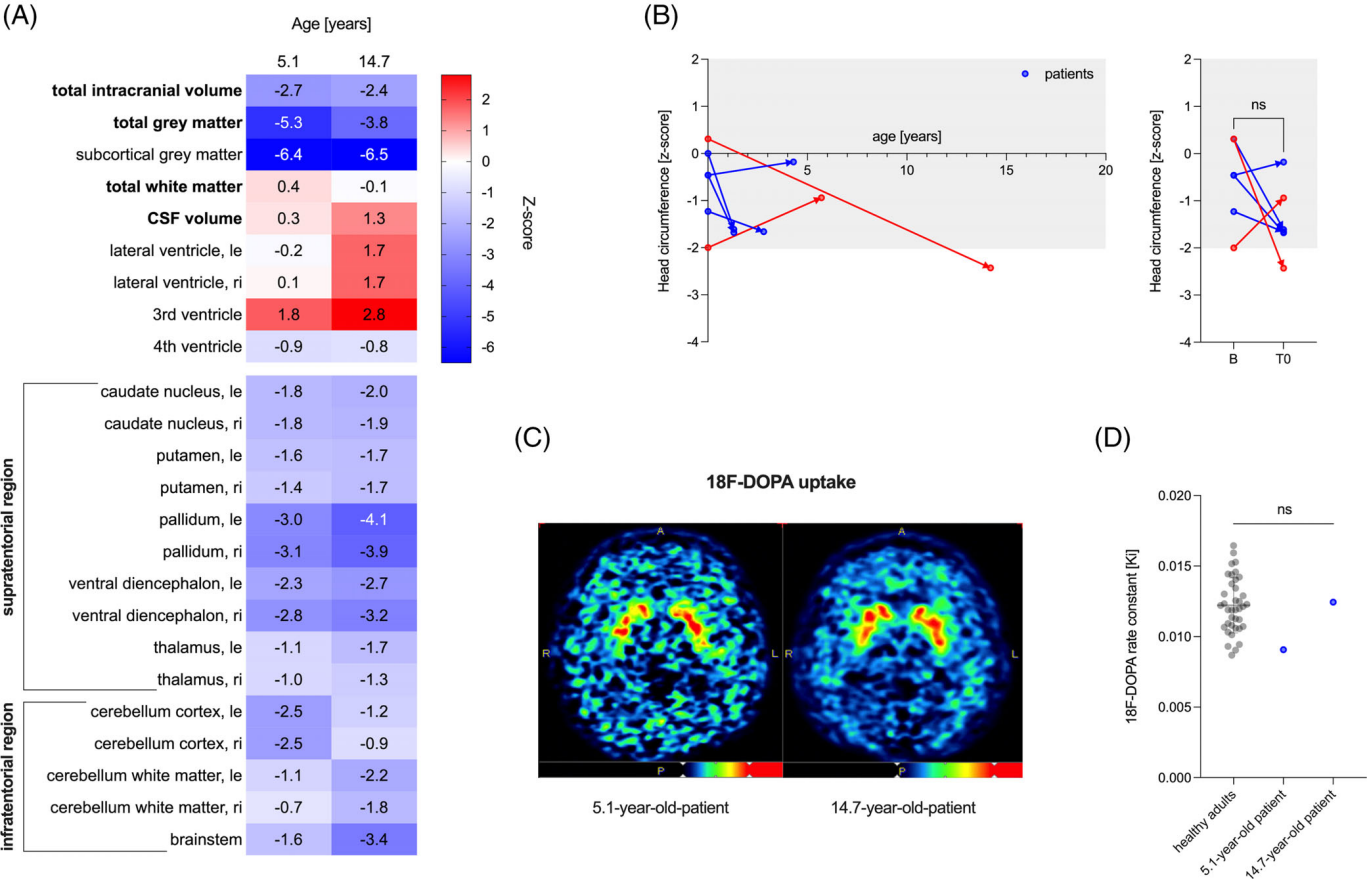
Neuroimaging findings. (**A**) Patients with MCT8 deficiency exhibited a reduction in total intracranial brain volume below two standard deviations (z scores, < −2.0z). This was particularly evident in the subcortical gray matter with a notable reduction in the size of the *Globus pallidus* (the site of dopamine action) and the ventral diencephalon, including the *Substantia nigra* (the site of dopamine synthesis). le, left; ri right. (**B**) Left panel: the z scores of the head circumferences (two‐point measurements) changed into both directions, n = 4 patients with decreasing z scores, n = 2 patients with increasing z scores. In terms of z scores, we did not see an overall significant change with progressing age. Additional data are required to distinguish between primary reduced brain volume and secondary brain atrophy in patients with MCT8 deficiency. The patients identified as 8 and 10, for which volumetric analyses were conducted, are indicated in red. The normality of the distribution was evaluated using the Shapiro–Wilk test (*α* = 0.05). The statistical significance of the differences between the groups was evaluated through the application of a paired *t*‐test. Right panel: (**B**) measurement of head circumference within the first week after birth; T0, measurement of head circumference before levodopa/carbidopa therapy. (**C**) For illustrative purposes, a non‐smoothed representative transversal image of the voxelwise modeled 18F‐DOPA rate constant (Ki) is presented. (**D**) The 18F‐DOPA uptake in the left caudate nucleus was found to be comparable to that observed in a previously published cohort of n = 44 healthy adults,^23^ indicating that this parameter may serve as a reliable indicator of nigrostriatal dopaminergic network integrity. The normality of the distribution was evaluated using the Shapiro–Wilk test (*α* = 0.05). The statistical significance of the differences between the groups was evaluated using an ordinary one‐way ANOVA test. The result was not statistically significant (ns). [Color figure can be viewed at wileyonlinelibrary.com]

We compared the segmented MRI brain volumes of patients 8 and 10 with previously published brain maps of 58,836 MRI scans from across the human lifespan^27^ (Fig. 4A). The results for both individuals were strikingly consistent. Total intracranial volume was reduced by more than two standard deviations (SD), with the most pronounced reduction in subcortical gray matter (−6.4 and −6.5 SD). In particular, (1) the *Globus pallidus*, as a major output structure of the basal ganglia and site of dopamine action, and (2) the ventral diencephalon, including the *Substantia nigra* with the dopaminergic neurons, were most affected by volume reduction (−2.4 to −4.1 SD). To differentiate between primary brain volume loss and secondary atrophy, we collected head circumference measurements in the first postnatal week and before initiation of l‐dopa/carbidopa treatment. Four of 6 patients had crossed percentiles with a reduction in head circumference, and the oldest patient 10 had developed secondary microcephaly at age 14.2 years (Fig. 4B). More data are needed to draw conclusions. In agreement with previously published data,^8^ we did not observe signal changes, including susceptibility‐weighted imaging (SWI) sequences, in the basal ganglia on MRI in all of 7 patients (data not shown). F18‐DOPA PET MRI, which assesses the functional integrity of nigrostriatal dopaminergic networks, appeared normal in patients 8 and 10 compared to adult controls (n = 44),^23^ as would be expected in monoamine biogenesis disorders other than aromatic l‐amino acid decarboxylase deficiency (Fig. 4C,D).^28^

## Discussion

With this retrospective study, we describe AHDS as a further rare disease that can be classified as “childhood parkinsonism.” In addition to the clinical signs of parkinsonism, the distinct constellation of biogenic amines with decreased HVA and HVA/5‐HIAA ratios in the cerebrospinal fluid suggests an isolated dopamine dyshomeostasis.^29^ This is supported by reduced subcortical gray matter volumes, especially of the Globus pallidus as a site of dopamine action and of the ventral diencephalon (including the Substantia nigra) as the site of dopamine synthesis. According to this hypothesis, substitution with l‐dopa/carbidopa led to a significant and long‐lasting improvement in patient‐oriented outcomes (improved axial tone and fine motor skills, reduced hypokinesia, rigidity, and dystonia). Only one patient did not benefit from therapy, but this was only tested for a (too) short period of 1 week. Underdosing (<10.0/2.5 mg/kg/day) or too short a treatment period may account for the lack of response in single case reports of l‐dopa/carbidopa supplementation.^8^, ^9^, ^24^

Infantile‐onset parkinsonism is an exceedingly rare and underdiagnosed condition. It has distinct features from Parkinson's disease (PD), one of the most common neurological disorders in the adult population. Whereas in PD, neurodegeneration of dopaminergic neurons in the *Pars compacta* of the *Substantia nigra* ^30^ leads to the cardinal signs of bradykinesia and tremor or rigidity,^31^ the dysfunction of dopamine action in the immature brain causes a more complex clinical picture. Because consensus clinical diagnostic criteria of pediatric‐onset parkinsonism have long been lacking, Leuzzi and colleagues recently proposed an initial classification for parkinsonism in childhood.^25^ These included (1) degenerative conditions with a monogenetic variant of PD and very early onset (juvenile parkinsonism and dystonia‐parkinsonism),^32^, ^33^ (2) acquired conditions leading to parkinsonism, for example, due to asphyxia, intoxication, or infection,^34^ (3) multisystem brain disorders with neurodegenerative or metabolic etiology (eg, Wilson's disease, Pantothenate kinase‐associated neurodegeneration), and (4) genetic disorders with generalized disruption of neurodevelopment (developmental parkinsonism, infantile degenerative parkinsonism, and parkinsonism in the setting of neurodevelopmental disorders). Although (1) juvenile parkinsonism and dystonia‐parkinsonism mimic PD with an earlier onset, children with conditions of the latter category (4) certainly present with different symptoms.^25^ Patients with developmental parkinsonism present with hypo‐, bradykinesia, rigidity, dystonia, inability to sit or stand, and global developmental delay.^25^ Additional clinical signs may include oculogyric crises, abnormal fetal movement patterns, oscillatory jerks, and periodic fluctuations.^25^ The pathophysiology is a selective, non‐degenerative derangement of dopaminergic connectivity, as seen in several neurotransmitter disorders (eg, tyrosine hydroxylase or aromatic l‐amino acid decarboxylase deficiency). If treatment is initiated early, patients can respond remarkably to neurotransmitter substitution.^25^ Regarding AHDS, we postulate that it can be classified as developmental parkinsonism (given the findings of reduced HVA in CSF and the favorable response to l‐dopa/carbidopa treatment) with some overlap to parkinsonism in the setting of neurodevelopmental disorders, where cognitive disability is the key finding while parkinsonism emerges later in life. Patients with AHDS showed only a partial response to l‐dopa/carbidopa substitution while the global developmental disorder persisted. This partial response to therapy may be due to the fact that, in addition to the functional impairment of dopamine synthesis, the patient's structural defect (reduced dopaminergic neurons and basal ganglia volume) may not be treatable by neurotransmitter substitution alone.

The observed structural abnormalities may be a consequence of prenatal MCT8 deficiency. This is supported by the fact that patients with the rather “historical” disease of “neurological type of cretinism,” who are affected by hypothyroidism *in utero* due to severe maternal iodine deficiency during pregnancy, present a clinical picture that resembles most features of childhood parkinsonism, including dystonia.^35^, ^36^, ^37^ In contrast, children with untreated congenital hypothyroidism due to agenesis of the thyroid gland show global developmental delay but no evidence of movement disorder.^38^ Whereas children with congenital hypothyroidism are hypothyroid only in the later stages of pregnancy and after birth due to their own impaired thyroid hormone synthesis, fetuses of iodine‐deficient mothers are exposed to hypothyroidism throughout gestation due to inadequate transplacental thyroid hormone supply. Thus, it seems likely that thyroid hormones play a critical role during the early period of neurodevelopment, including the formation of dopaminergic circuits, which may not be fully rescued by l‐dopa/carbidopa substitution after birth. This thesis is supported by the results of an off‐label trial in which a fetus with pathogenic *SLC16A2* mutation of an index family was treated prenatally by intra‐amniotic instillation of levothyroxine from 18 weeks of gestation until birth and subsequently developed significantly better than his brother, who was severely affected and about 2 years older.^39^

At the molecular level, we hypothesize that MCT8 deficiency disrupts thyroid hormone transport to neuronal target cells, resulting in altered gene transcription patterns *via* thyroid hormone receptors that act as transcription factors. In mice, more than 1000 genes are regulated by T3, and T3‐dependent genes (eg, *sonic hedgehog*, *orthodenticle homeobox 2*) play important roles in neurodevelopmental processes such as cell proliferation, cell fate decision, axonogenesis, synaptogenesis, and myelinogenesis.^40^ The consequences of thyroid hormone deficiency on the developing human brain are not yet known and are currently being investigated in human disease models such as human forebrain organoids.^41^ These emerging data already suggest a critical role for MCT8 in cerebral cortex development and regulation of multiple genes beyond the dopamine pathways, which may explain the only partial response to therapy in our patients.

Regarding the effect of MCT8 deficiency on dopamine action, Hassan and colleagues have shown *in vitro* that a hypothyroid condition inhibits the tyrosine hydroxylase as the rate‐limiting enzyme of dopamine synthesis,^42^ whereas, inversely, hyperthyroid states lead to an increase in dopamine metabolism.^43^ Whether this is also true for MCT8 deficiency remains to be determined in future studies. Given the species differences and the numerous limitations of the Mct8‐deficient mouse model, which does not resemble the human phenotype of patients, we would suggest the use of human iPSC‐derived dopaminergic midbrain neurons expressing MCT8^44^ as a future *in vitro* experimental system to address these important questions of the pathogenesis of parkinsonism in AHDS.^45^

Overall, our data establish AHDS as another syndrome that fulfills the clinical criteria of parkinsonism in childhood. Although there is no definitive treatment for the underlying MCT8 deficiency while gene therapies are still under development,^13^, ^14^ patients appear to benefit from symptomatic treatment with l‐dopa/carbidopa.

Limitations: This study is an initial observation in a small cohort of patients who had been entered into a dedicated patient registry. The initial observation of the positive effect of l‐dopa/carbidopa treatment has to be confirmed by a formal prospective, controlled blinded, cross‐over phase II/III trial.

## Author Roles

(1) Research Project: A. Conception, B. Organization, C. Execution; (2) Statistical Analysis: A. Design, B. Execution, C. Review and Critique; (3) Manuscript Preparation: A. Writing of the First Draft, B. Review and Critique.

N.M.W.: 1A, 1B, 1C, 2A, 2B, 2C, 3A, 3B

A.L.H.: 1A, 1B, 1C, 3B

R.P.: 1A, 1B, 3B

M.T.H.: 1B, 1C, 3B

A.D.O.: 1B, 1C, 2A, 2B, 3B

M.B.: 1B, 1C, 2A, 2B, 3B

C.G.: 1B, 1C, 3B

S.M.: 1C, 2A, 3B

M.W.: 1B, 1C, 3B

A.Z.: 1B, 3B

A.P.: 1B, 3B

C.R.: 1B, 1C, 3B

C.L.: 1B, 1C, 3B

C.F.: 1B, 1C, 3B

K.B.: 1B, 1C, 3B

S.J.K.: 1B, 1C, 3B

S.C.: 1B, 1C, 3B

A.M.K.: 1B, 3B

A.T.: 1A, 1B, 1C, 2C, 3B

H.K.: 1A, 2C, 3A, 3B

T.O.: 1A, 1B, 1C, 2C, 3B

M.S.: 1A, 1B, 1C, 2A, 2B, 2C, 3A, 3B

## Institutional Review Board Statement

Ethical approval for the study was obtained from the Institutional Review Board of Charité‐Universitätsmedizin Berlin, Campus Virchow Klinikum in the year 2020 (EA2/026/20). The study was conducted in accordance with the tenets of the Declaration of Helsinki.

## Supporting information


**Data S1.** Supplementary Methods 1: The Infantile Parkinsonism‐Dystonia Rating Scale (IPDRS).

**Data S2.** Supplementary Methods 2: Neuroimaging, volumetry, and positron emission tomography (PET) studies.

**Figure S1.** Development and quality of life under levodopa/carbidopa treatment.
(**A–E**) Patients exhibited significant improvement in fine motor skills when assessed using the Bayley Scales of Infant and Toddler Development Third Edition (BSID‐III), whereas no notable changes were observed in other categories. The natural history data were extracted with ImageJ from the published work referenced in the text.^11^ The hypothesis of a normal distribution was always tested using the Shapiro–Wilk test (*α* = 0.05). In the event that the data were normally distributed, the statistical significance of the differences between the groups was evaluated through the application of a paired *t*‐test. In the event that the data were not normally distributed, the Wilcoxon test was applied. ns, not significant; *P ≤ 0.05. (**F**) No significant improvement in the quality of life, as measured by the Cerebral Palsy Child Health Index of Life with Disabilities (CPCHILD), was reported. One family experienced a period during which a gastric tube was placed in their child and hip surgery was performed, and thus reported a reduced quality of life. The data were not normally distributed according to the Shapiro–Wilk test. The statistical significance of differences between the groups was tested by applying the Wilcoxon test. ns, not significant.

**Figure S2.** No adverse drug reactions under levodopa/carbidopa treatment.
Adverse drug reactions of the levodopa/carbidopa treatment were neither reported by parents (**A–F**) nor could any significant negative changes in patients' (depicted in blue) body mass index (BMI), heart rate, or laboratory tests be identified. Three patients were fitted with a gastric tube (depicted in orange). The natural history data (depicted in gray) were extracted from published work using the image processing software ImageJ. The hypothesis of a normal distribution was tested with the Shapiro–Wilk test (*α* = 0.05). If data were normally distributed, the statistical significance of the differences between the groups was evaluated using a paired *t*‐test. Conversely, in instances where the data were not normally distributed, the Wilcoxon test was employed. ns, not significant; *P ≤ 0.05; fT3, free triiodothyronine; fT4, free thyroxine; TSH, thyroid stimulating hormone; bpm, beats per minute; CK, creatine kinase (as a marker for muscle fiber damage); ALT, alanine transaminase (as a marker for liver function damage).

## Data Availability

The data that support the findings of this study are available from the corresponding author upon reasonable request.
