# Supplementary material for: Patients with Allan‐Herndon‐Dudley Syndrome (MCT8 Deficiency) Display Symptoms of Parkinsonism in Childhood and Respond to Levodopa/Carbidopa Treatment
Source: Mov Disord. 2025 Mar 15;40(5):938–49. doi: 10.1002/mds.30152 (PMC12089910; doi:10.1002/mds.30152)
Supplement: Supplementary file 1 — Data S1. Supplementary Methods 1: The Infantile Parkinsonism‐Dystonia Rating Scale (IPDRS). [file MDS-40-938-s001.docx]

**Supplementary methods 1: *Infantile Parkinsonism-Dystonia Rating Scale (IPDRS)*.**

*by Pons R, Pearson TS, Perez-Dueñas B, Garcia-Cazorla A, Kurian MA, Dalibigka, Z, Outsika C,Kokkinou E, Zouvelou B, Singatulina M, Darling A, O’Callaghan M, Spaull R, Steel DBD , Forjaz MJ, Rodriguez-Blazquez C*

The IPDRS will soon be submitted to *Movements Disorders* and includes the following subscales:

1. **Non-motor symptoms** (caregiver report)
   - Autonomic dysfunction 5 items

*Thermoregulation, Respiratory, Gastrointestinal, Sleep, Other*

- - Mood dysfunction / emotional lability 1 item

1. **Motor symptoms** (physical examination, caregiver report for OGC)
   - Oculogyric crises (OGC) (severity, duration, frequency) 3 items
   - Bradykinesia 5 items

*Spontaneous movements (global, facial, lower limbs, upper limbs)*

*Voluntary movements (upper limbs)*

- - Tremor (distribution, severity) 2 items
  - Rigidity (distribution, severity) 2 items
  - Dystonia (severity) 4 items

*Facial, axial, upper limbs, lower limbs*

- - Axial hypotonia 2 items
  - Motor developmental delay 1 item

1. **Dyskinesia** (physical examination)
   - Hyperkinetic involuntary movements (severity, duration, distribution) 3 items

*(excludes dystonia, tremor, tics, stereotypies)*
