# Supplementary material for: Patients with Allan‐Herndon‐Dudley Syndrome (MCT8 Deficiency) Display Symptoms of Parkinsonism in Childhood and Respond to Levodopa/Carbidopa Treatment
Source: Mov Disord. 2025 Mar 15;40(5):938–49. doi: 10.1002/mds.30152 (PMC12089910; doi:10.1002/mds.30152)
Supplement: Supplementary file 2 — Data S2. Supplementary Methods 2: Neuroimaging, volumetry, and positron emission tomography (PET) studies. [file MDS-40-938-s002.docx]

**Supplementary methods 2: Neuroimaging, volumetry, and PET-studies**

Neuroimaging data were collected retrospectively. For volumetric neuroimaging, magnetic resonance imaging (MRI) was performed on a 3 Tesla MRI system (Skyra Magnetom, Siemens Healthineers, Erlangen, Germany) with a 64-element head coil. The sagittal T_1_-weighted magnetization prepared rapid gradient echo (MPRAGE) sequence took 5 min, 21 s (voxel size 0.9 × 0.9 × 0.9 mm^3^, field of view 240 mm^2^, repetition time [TR] = 2,300 ms, echo time [TE] = 2.32 ms, TI = 900 ms, flip angle 1 = 8°). Digital imaging and communications in medicine (DICOM) data were converted to the Neuroimaging Informatics Technology Initiative (NIfTI) format using the dcm2niix tool.[^1^](https://www.zotero.org/google-docs/?jksnys) The resulting NIfTI files were segmented according to the Desikan-Killiany atlas[^2^](https://www.zotero.org/google-docs/?es8MT4) using the Freesurfer v6.0 recon-all pipeline.[^3^](https://www.zotero.org/google-docs/?broken=EHX484) The resulting segmentation masks were compared with those of healthy subjects.[^3^](https://www.zotero.org/google-docs/?G0eMAV) Total white matter brain volume was calculated as follows: total white matter = supra tentorial volume excluding ventricles - (total gray matter + left cerebellar cortex + right cerebellar cortex)/3.

Positron emission tomography (PET) list-mode data were acquired in a Biograph mMR PET/MRI scanner (Siemens Healthineers, Erlangen, Germany) over a 60 minute period, initiated concurrently with intravenous bolus administration of 86 MBq (14-year-old patient) and 49 MBq (5-year-old patient) 18F-DOPA. List-mode data were reconstructed with standard parameters (OSEM, 3 iterations, 21 subsets, correction for attenuation and scatter) and in 20 frames (3 x 20 s, 3 x 1 min, 3 x 2 min, 3 x 3 min, 7 x 5 min, 1 x 6 min). PET data were analyzed using Statistical Parametric Mapping 12 (Welcome Department of Imaging Neuroscience, Institute of Neurology, London, <http://www.fil.ion.ucl.ac.uk/spm/>) and based on a previously described pipeline.[^4^](https://www.zotero.org/google-docs/?0FWJ9y) Normal values of n=44 adult 18F-DOPA rate constants (Ki) in regions of interest were digitized from published figures.[^5^](https://www.zotero.org/google-docs/?L2A44u) PET was corrected for head motion between frames and the individual PET mean images and individual T1 images were co-registered. Each T1 image was spatially normalized using the unified segmentation approach with default setting and the computed normalization parameters were then applied to the co-registered PET frames and the individual subcortical brain segmentation masks from FSL. Dopamine synthesis capacity was quantified as 18F-DOPA rate constant per min (Ki), which was estimated voxel by voxel using Gjedde-Patlak linear graphical analysis.[^5^](https://www.zotero.org/google-docs/?ap7kpR) Radioactivity time curves in the cerebellum mask from the automated anatomical labeling atlas were used as input function.[^6^](https://www.zotero.org/google-docs/?K2FM0w) The linear fit was restricted to the time interval 5-60 min after injection. Mean Ki values were extracted from the voxelwise map for each individual subcortical regions-of-interest (caudate nucleus, putamen, accumbens nucleus, pallidum, amygdala, hippocampus, thalamus).

## Supplementary References

[1. Li X, Morgan PS, Ashburner J, Smith J, Rorden C. The first step for neuroimaging data analysis: DICOM to NIfTI conversion. J Neurosci Methods 2016;264:47–56.](https://www.zotero.org/google-docs/?e7WeZK)

[2. Desikan RS, Ségonne F, Fischl B, et al. An automated labeling system for subdividing the human cerebral cortex on MRI scans into gyral based regions of interest. NeuroImage 2006;31(3):968–80.](https://www.zotero.org/google-docs/?e7WeZK)

[3. Rutherford S, Fraza C, Dinga R, et al. Charting brain growth and aging at high spatial precision. eLife 2022;11:e72904.](https://www.zotero.org/google-docs/?e7WeZK)

[4. Deserno L, Huys QJM, Boehme R, et al. Ventral striatal dopamine reflects behavioral and neural signatures of model-based control during sequential decision making. Proc Natl Acad Sci U S A 2015;112(5):1595–600.](https://www.zotero.org/google-docs/?e7WeZK)

[5. Lorenz RC, Gleich T, Buchert R, Schlagenhauf F, Kühn S, Gallinat J. Interactions between glutamate, dopamine, and the neuronal signature of response inhibition in the human striatum. Hum Brain Mapp 2015;36(10):4031–40.](https://www.zotero.org/google-docs/?e7WeZK)

[6. Tzourio-Mazoyer N, Landeau B, Papathanassiou D, et al. Automated Anatomical Labeling of Activations in SPM Using a Macroscopic Anatomical Parcellation of the MNI MRI Single-Subject Brain. NeuroImage 2002;15(1):273–89.](https://www.zotero.org/google-docs/?e7WeZK)
