# Supplementary figures and images for: Patients with Allan‐Herndon‐Dudley Syndrome (MCT8 Deficiency) Display Symptoms of Parkinsonism in Childhood and Respond to Levodopa/Carbidopa Treatment
Source: Mov Disord. 2025 Mar 15;40(5):938–49. doi: 10.1002/mds.30152 (PMC12089910; doi:10.1002/mds.30152)

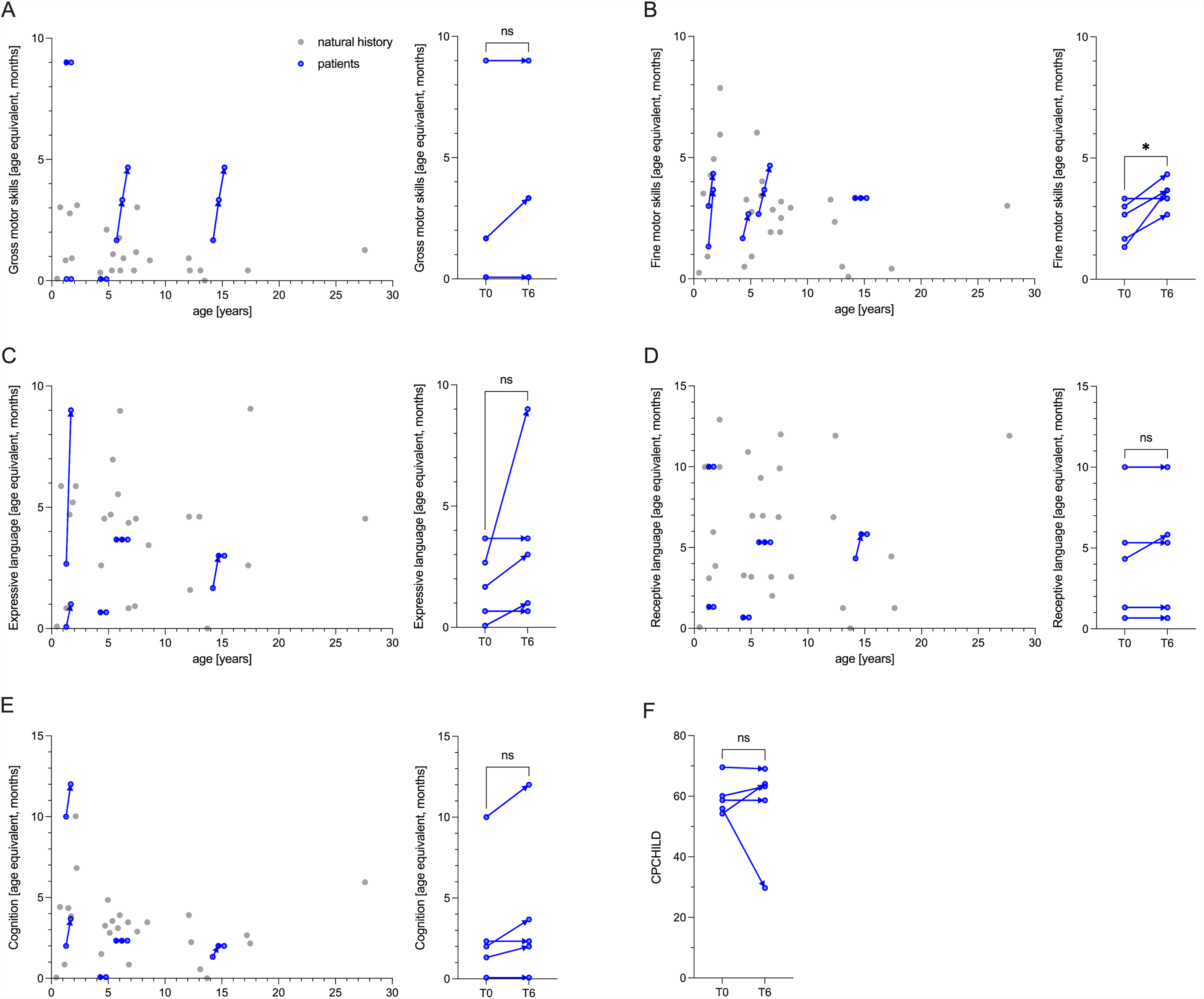

Supplement: Supplementary file 3 — Figure S1. Development and quality of life under levodopa/carbidopa treatment. (A–E) Patients exhibited significant improvement in fine motor skills when assessed using the Bayley Scales of Infant and Toddler Development Third Edition (BSID‐III), whereas no notable changes were observed in other categories. The natural history data were extracted with ImageJ from the published work referenced in the text. 11 The hypothesis of a normal distribution was always tested using the Shapiro–Wilk test (α = 0.05). In the event that the data were normally distributed, the statistical significance of the differences between the groups was evaluated through the application of a paired t‐test. In the event that the data were not normally distributed, the Wilcoxon test was applied. ns, not significant; *P ≤ 0.05. (F) No significant improvement in the quality of life, as measured by the Cerebral Palsy Child Health Index of Life with Disabilities (CPCHILD), was reported. One family experienced a period during which a gastric tube was placed in their child and hip surgery was performed, and thus reported a reduced quality of life. The data were not normally distributed according to the Shapiro–Wilk test. The statistical significance of differences between the groups was tested by applying the Wilcoxon test. ns, not significant. [file MDS-40-938-s003.tiff]

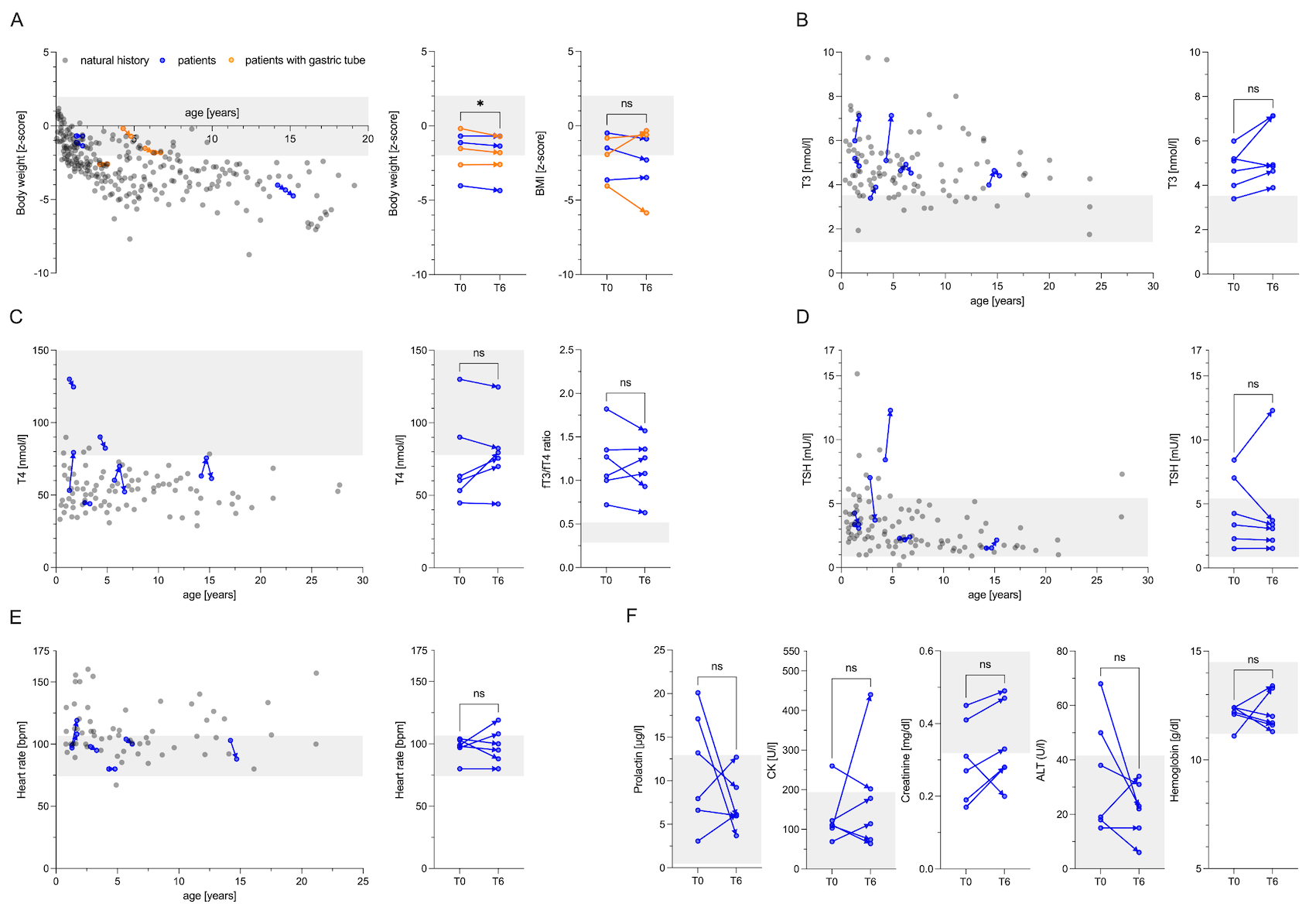

Supplement: Supplementary file 4 — Figure S2. No adverse drug reactions under levodopa/carbidopa treatment. Adverse drug reactions of the levodopa/carbidopa treatment were neither reported by parents (A–F) nor could any significant negative changes in patients' (depicted in blue) body mass index (BMI), heart rate, or laboratory tests be identified. Three patients were fitted with a gastric tube (depicted in orange). The natural history data (depicted in gray) were extracted from published work using the image processing software ImageJ. The hypothesis of a normal distribution was tested with the Shapiro–Wilk test (α = 0.05). If data were normally distributed, the statistical significance of the differences between the groups was evaluated using a paired t‐test. Conversely, in instances where the data were not normally distributed, the Wilcoxon test was employed. ns, not significant; *P ≤ 0.05; fT3, free triiodothyronine; fT4, free thyroxine; TSH, thyroid stimulating hormone; bpm, beats per minute; CK, creatine kinase (as a marker for muscle fiber damage); ALT, alanine transaminase (as a marker for liver function damage). [file MDS-40-938-s004.tiff]
